# Acceptability of a Mobile Phone Support Tool (Call for Life Uganda) for Promoting Adherence to Antiretroviral Therapy Among Young Adults in a Randomized Controlled Trial: Exploratory Qualitative Study

**DOI:** 10.2196/17418

**Published:** 2021-06-14

**Authors:** Adelline Twimukye, Agnes Bwanika Naggirinya, Rosalind Parkes-Ratanshi, Ronnie Kasirye, Agnes Kiragga, Barbara Castelnuovo, Jacob Wasswa, Maria Sarah Nabaggala, Elly Katabira, Mohammed Lamorde, Rachel Lisa King

**Affiliations:** 1 Infectious Diseases Institute College of Health Sciences Makerere University Kampala Uganda; 2 Department of Public Health & Primary Care Institute of Public Health University of Cambridge Cambridge United Kingdom; 3 College of Health Sciences School of Medicine Makerere University Kampala Uganda; 4 Institute for Global Health Sciences University of California San Francisco, CA United States

**Keywords:** HIV, mHealth, young adults, adherence, qualitative, Uganda

## Abstract

**Background:**

Adherence to treatment is critical for successful treatment outcomes. Although factors influencing antiretroviral therapy (ART) adherence vary, young adults are less likely to adhere owing to psychosocial issues such as stigma, ART-related side effects, and a lack of access to treatment. The Call for Life Uganda (CFLU) mobile health (mHealth) tool is a mobile phone–based technology that provides text messages or interactive voice response functionalities through a web interface and offers 4 modules of support.

**Objective:**

This study aims to describe the acceptability and feasibility of a mobile phone support tool to promote adherence to ART among young adults in a randomized controlled trial.

**Methods:**

An exploratory qualitative design with a phenomenological approach at 2 study sites was used. A total of 17 purposively selected young adults with HIV infection who had used the mHealth tool CFLU from 2 clinics were included. In total, 11 in-depth interviews and 1 focus group discussion were conducted to examine the following topics: experience with the CFLU tool (benefits and challenges), components of the tool, the efficiency of the system (level of comfort, ease, or difficulty in using the system), how CFLU resolved adherence challenges, and suggestions to improve CFLU. Participants belonged to 4 categories of interest: young adults on ART for the prevention of mother-to-child transmission, young adults switching to or on the second-line ART, positive partners in an HIV-discordant relationship, and young adults initiating the first-line ART. All young adults had 12 months of daily experience using the tool. Data were analyzed using NVivo version 11 software (QSR International Limited) based on a thematic approach.

**Results:**

The CFLU mHealth tool was perceived as an acceptable intervention; young adults reported improvement in medication adherence, strengthened clinician-patient relationships, and increased health knowledge from health tips. Appointment reminders and symptom reporting were singled out as beneficial and helped to address the problems of forgetfulness and stigma-related issues. HIV-related stigma was reported by a few young people. Participants requested extra support for scaling up CFLU to make it more youth friendly. Improving the tool to reduce technical issues, including network outages and a period of software failure, was suggested. They suggested that in addition to digital solutions, other support, including the promotion of peer support meetings and the establishment of a designated space and staff members for youth, was also important.

**Conclusions:**

This mHealth tool was an acceptable and feasible strategy for improving ART adherence and retention among young adults in resource-limited settings.

**Trial Registration:**

ClinicalTrials.gov NCT02953080; https://clinicaltrials.gov/ct2/show/NCT02953080

## Introduction

### Background

In 2018, of the 3.9 million young people living with HIV globally, 80% were in Sub-Saharan Africa [1]. The 2017 Uganda Bureau of Statistics report indicated that more than half of Uganda’s population (55%) is aged under 18 years [2]. The burden of HIV among youth in Uganda is high. In Uganda, HIV prevalence triples from those aged 15-19 years (1.1%) to those aged 20-24 years (3.3%), suggesting that new infections remain an issue in this age group [3]. Among young adults (aged 15-24 years), there is a disparity in HIV prevalence by sex. HIV prevalence is almost 4 times higher among women than men aged 15-19 years and 20-24 years [4].

HIV care and treatment services in the region should adapt to adequately meet the antiretroviral therapy (ART) demand to suppress the viral load to undetectable levels and improve health outcomes. Suboptimal ART adherence over time predicts virological failure, development of drug resistance, and death [5-8]. Adolescents and young adults have poorer rates of adherence to ART and retention in care than older adults [9]. Young adults are less likely to adhere to ART medication if they have psychosocial issues such as stigma, previously experienced side effects with the treatment, and lack of access to treatment [10-12].

In 2018, 50% of the population (24.7 million) were phone subscribers (fixed or cellular) [13]. Compared with adults (40%), young adults (66%) are more likely to use mobile phone SMS text messaging [13]. Mobile phones have become a common accessory for most young people in Uganda. Self-reported quantitative survey data collected in 2008-2009 from 1503 secondary school students in Mbarara, Uganda, suggest that 27% currently had mobile phones and about half (51%) of all students and 61% of those who owned a mobile phone believed that they would access an SMS text messaging–based HIV prevention program [14]. One study assessed the patterns and dynamics of mobile phone use among an ART cohort in rural Uganda, ascertained its feasibility for improving clinic attendance, and found that mobile phones have the potential for use in resource-constrained settings to improve the clinical management of people living with HIV [15]. Texting-based and other mobile health (mHealth) interventions could be particularly suitable for young adults who, irrespective of their socioeconomic status, are typically competent users of mobile phones and text messages. Globally, mHealth interventions have resulted in improvements in treatment adherence for patients with asthma, diabetes, and tuberculosis [16].

### Objectives

Various mHealth studies have shown improved virological outcomes [17,18], lower risk of nonadherence [19-21], and good retention in care before ART initiation [15]. However, there is little specific evidence on the effect of mHealth on young adults living with HIV. We used qualitative methods to describe the acceptability of using Call for Life Uganda (CFLU, an adherence support tool offering interactive voice response [IVR] or SMS) to promote adherence to ART for young adults in a randomized controlled trial (RCT).

## Methods

### Study Design

We implemented an exploratory qualitative design using a phenomenological approach to describe the acceptability and feasibility of a mobile phone support tool to promote adherence to ART among young adults. The study was nested within an open-label RCT titled *Improving Outcomes in HIV Patients Using Mobile Phone–Based Interactive Software Support*. The technology evaluated in this study is connect for life (CfL), a MOTECH-based, open-source platform developed by the Grameen Foundation and the University of Southern Maine. It was supported by Janssen, the Pharmaceutical Companies of Johnson and Johnson, and was released under the terms of MOTECH’s open-source license agreement. We adapted CfL for use among people living with HIV and named the system CFLU. CFLU allows a computer to interact with patients using voice and tone input via keypad (IVR) or by SMS, with the choice made by the patient. All content was developed in English and 2 additional local languages, following the Uganda HIV treatment guidelines. People living with HIV in the control arm received standard of care comprising face-to-face facility appointments, no remote adherence or appointment reminders, and no remote symptom reporting. In the intervention arm, the people living with HIV received standard of care and daily adherence IVR call or SMS text messaging at the preplanned times of taking ART. In the intervention arm, the people living with HIV also received a dedicated call offering a health education message (a health tip) weekly. Patients received IVR calls or SMS text message appointment reminders on or before the scheduled appointment date. The CFLU platform allows patients to report symptoms at the end of the scheduled call, or at any time, through a toll-free line. Multimedia Appendix 1 illustrates the call flow from the CFLU tool to the end user. Participants chose the preferred languages, time, and frequency of receiving calls.

In this qualitative study, we used in-depth interviews (IDIs) and 1 focus group discussion (FGD) to gain insights into individual and community perspectives about the CFLU tool. The purpose of the FGD in this study was to stimulate group conversations on specific themes, to assess the differences and similarities in perceptions, values, norms, and preferences among young adults. We explored only the normative and topics that were not considered sensitive in the FGD [22].

### Study Participants and Selection

Participants were enrolled from 2 HIV clinics acting as study sites for the CFLU RCT (the Infectious Diseases Institute clinic at Mulago National Referral Hospital, Kampala, and Kasangati Health Centre facility level IV, a periurban government health facility). Purposive sampling of young adults with HIV infection aged 18-25 years was carried out from these 2 clinics.

The total number of young adults was 161. We undertook purposive sampling within this group to recruit them for this study. Inclusion criteria were registration in the CfL RCT, had access to a mobile phone, had the ability to use basic mobile phone functions such as making and receiving calls, and willingness to comply with scheduled visits. Patients who understood 1 of the 2 local languages or English were eligible for the study. Young adults who were critically ill; those aged under 18 years; and those whose clinical condition interfered with the appropriate use of their mobile phone, such as deafness or severe cognitive impairment, were excluded from the study. The young adults could also belong to other groups of interest enrolled in the study, including pregnant women receiving ART for the prevention of mother-to-child transmission, those switching to or on second-line ART, those initiating first-line ART, and positive partners in a discordant relationship.

### Data Collection

We made telephone calls to potential young adults to brief them about the study and invite them for the study. We negotiated an appropriate time, place, and day for the interview with the IDI and FGD participants and booked secure private locations to conduct interviews at the Infectious Diseases Institute clinic and Kasangati, as appropriate. Clinic study staff, a counselor with social science expertise and Good Clinical Practice, and a postdoctoral scholar trained in FGD note taking collected the data. The FGD had a range of 6-12 participants. The clinic study staff contacted potential participants by phone, described the study, and extended an invitation to participate. We conducted 2 interviews in English and 10 in the local language (Luganda) at the Infectious Diseases Institute and Kasangati in private locations (IDIs) and community centers (FGDs). Before data collection, we translated the topic guides from English into Luganda, the main language spoken in the catchment area of the study clinics. Each IDI and FGD lasted for approximately 1 hour and were audio recorded and complemented by written notes. We examined the following topics in IDIs and FGD: (1) experience with the CFLU tool (likes and dislikes), (2) components of the tool (health tips, pill and appointment reminders, and symptom reporting), (3) efficiency and willingness to pay for the system (level of comfort, ease, or difficulty in using the system), (4) how CFLU resolved adherence challenges, and (5) suggestions to improve CFLU. All young adults had 12 months of daily experience using the tool. Young adults were recruited and interviewed until no new themes emerged, and saturation was reached when there was repetition of the previously mentioned themes.

### Data Analysis and Interpretation

All audio recordings from IDIs and FGDs were transcribed verbatim after translation by independent professionals. The research team read the typed transcripts several times and filled in the gaps by listening to the audio recordings. Data were managed and analyzed using NVivo version 11 (QSR International Limited). Each independent transcript was read and reread by a senior social scientist for emergent themes and recurrent ideas and then aggregated into themes. Codes were assigned to relevant segments of the text; similar codes were aggregated to form themes, which were then used to address the research questions and develop coherent narratives. The team developed an explicit codebook describing each category and theme. In the next step, the team sorted the quotes based on themes. The team then examined the degree to which these themes were distributed across gender, age group, and social target group. After team members read the interview transcripts, data were coded into meaning units and major themes were developed. Quotations and key phrases are highlighted in the findings.

### Ethical and Regulatory Approval

The CFLU RCT was approved by the Makerere University School of Public Health Higher Degrees Research and Ethics and Committee (Number 378) and the Uganda National Council of Science and Technology (Number Health Sciences 3005) and was registered with ClinicalTrials.gov (NCT02953080). All the study procedures, compensation, benefits, potential risk of participation, and voluntary and confidential nature of participation were discussed. Written informed consent was obtained from all respondents before enrollment in the qualitative study. For young adults with low literacy, we used a thumbprint in the presence of a witness.

## Results

### Overview

A total of 82 young adults who used the CFLU tool were given a telephone call, inviting them for FGD or IDIs (Multimedia Appendix 2). Of the 82 young adults, 37 were found ineligible as their age limit was slightly more than that required for young adults (26-28 years) and 28 could not make it for the discussion because of various reasons such as busy schedules and phones not available owing to poor network. A total of 21 young adults agreed to participate, 17 on the intervention arm and 4 on the control arm (analyzed separately and not included here (Multimedia Appendix 2).

A total of 11 IDIs and 1 FGD with those on the intervention arm were conducted. A total of 6 young adults were from the Infectious Diseases Clinic, whereas 11 were from Kasangati Health Center IV. The age range of young adults was 18-25 years; the majority were females, and more than 81% (17/21) of them were in a steady sexual relationship. Most young adults had a formal education, with only 14% (3/21) having reached the tertiary level (Table 1).

The results covered 4 major themes related to CFLU. These were attitudes toward CFLU, components of CFLU, barriers to and challenges of CFLU, and suggestions or recommendations from youth to improve CFLU. The following sections organize the results into 3 levels: personal and household factors, facility or service delivery factors, and community factors (Table 2).

**Table 1:** Demographics of participants who took part in the qualitative study (N=21)^a^.

Population demographics			Participants, n (%)
**Age (years)**			
	18-19	3 (14)	
	20-24	18 (85)	
**Gender**			
	Male	5 (24)	
	Female	16 (76)	
**Marital status**			
	In a relationship	17 (81)	
	Single	4 (19)	
**Education status**			
	Secondary education and primary education	15 (71)	
	Tertiary education	3 (14)	
	No formal education	1 (5)	
	Missing information	2 (10)	

^a^Four young adults were excluded because they had no experience with the tool.

**Table 2:** Frequency of each theme for young adults on CFLU^a^ (N=21).

Theme and categories		Patients, n (%)	Number of responses
**Positive perceptions toward the CFLU^a^ tool**			
	CFLU system perceived as an adherence supporter	6 (29)	18
	Management of HIV stigma–related challenges	6 (29)	18
	Increased psychosocial support	10 (48)	25
	Strengthened clinician-patient relationship	5 (24)	7
	The kind tone of voice of the system	3 (14)	6
	Confidentiality and privacy	9 (43)	13
	Ease of system use	7 (33)	11
**Components of the CFLU tool**			
	Clinic visit call reminders	12 (57)	34
	Symptoms reporting	12 (57)	46
	Health tips call	12 (57)	44
	Daily pill reminder call	12 (57)	57
**Barriers to and challenges of the CFLU tool**			
	Technical issues with the system	11 (52)	22
	Poor access to mobile phones	12 (57)	23
	Fear of HIV-related stigma	6 (29)	18
**Suggestions to improve the CFLU tool**			
	Resolving adherence challenges	5 (24)	9
	Suggesting additional health tips	12 (57)	45
	Providing adolescent peer support	7 (33)	20
	Scaling up CFLU	8 (38)	11
	Sensitizing about CFLU and HIV	9 (43)	16
	Making CFLU appealing	2 (10)	8
	Getting the designated space for CFLU	1 (5)	3
	Getting the designated staff for CFLU	5 (24)	10
	Resolving the stigma	4 (19)	6
	Combining the CFLU appointment with routine clinic appointment	31 (4)	5
	Other suggestions	4 (19)	4

^a^CFLU: Call for Life Uganda.

### Positive Perceptions Toward CFLU

#### Personal and Household Related

##### CFLU System Perceived as an Adherence Supporter

Most young adults from both sites, but mainly from Kasangati, perceived the CFLU system as an aid to adherence through its pill reminders, especially for youth who had trouble taking their medication and who forgot to take their medication because of busy work and school schedules.

It gives me strength, when I had just started my treatment I was very scared. So, I didn’t know...how to take my medicine....The pill was very large...but the way the answering machine speaks, it gives me courage to really take my pills.

FGD, female, 18-24 years, intervention, Kasangati

CFLU comes in as a treatment supporter to remind one to take medication; it is like a parent asking, “Hey, did you swallow your medication?” It is a voice message but sounds like a person who is aware of one’s HIV status.

##### Resolving Forgetfulness

Both male and female youth across different groups admitted to poor adherence to ART in the past and believed that CFLU would resolve most of the barriers to adherence, such as forgetfulness. The reminder call was deemed critical when a young adult slept off or during a busy school or work schedule:

I used to forget to take pills on time. But the doctor cares so much and calls to remind me to take my medication...majority miss taking drugs but each time a call comes through, it reminds you, most youth get tired mentally; they forget.

IDI, 18 years, male, intervention, Kasangati

CFLU is good because it helps me take my pills on time, whenever I am very tired and fatigued; I sleep off before the time for taking pills... when my phone rings, I wake up immediately, and straight away I take my drugs.

FGD, female, 18-24 years, intervention Kasangati

There was a perception among most young adults from both sites that the tool improved their health through viral load suppression. Most youth on CFLU said that pill reminders helped them take drugs properly, daily, and on time, which led to viral suppression compared with when they were not on the system:

It’s helpful to improve pill adherence & keeping appointments [that] will cause improvement of health, if the viral load is reducing then the opportunistic infections are reducing.

IDI, female, 24 years, intervention arm, Mulago

##### Management of HIV Stigma–Related Challenges

Most young adults, mainly from Infectious Diseases Institute clinic, stated that CFLU resolved the fear of stigma through its health tips that continuously educated them about how to overcome stigma and emphasized the importance of disclosure to avoid stigma and the importance of adherence to ART. Adherence to ART was easier in the context of young adults on CFLU who had not disclosed their HIV status:

Call for life does continuous education about overcoming stigma. It advises and teaches us to overcome stigma which encourages us in our HIV situation, we (youth) cease to be fearful.

IDI, 24 years, intervention, Mulago

We follow positive living health tips, they tell us “you don’t have to worry about your status when you are HIV positive” you know we are always worried, some of us have stigma. They just teach us to stay and live positive, eat a balance diet.

FGD, female, 18-24 years, intervention arm, Kasangati

The fact that we are not supposed to talk back after receiving the call, sometimes when we are in public places, we do not have to worry that someone will hear what you are saying you just listen and keep following the prompts and others will not know anything. It works for me personally.

FGD, female, 18-24 years, intervention arm, Kasangati

##### Increased Psychosocial Support

Most young adults, mainly from Kasangati, expressed that they received psychosocial support from the tool, especially in those starting ART. Emotionally, the youth on CFLU felt that they were not alone. Through interaction with the loving voice that instructed them on how to take treatment, they were able to obtain coping support and gain hope for the future. The counseling provided during CFLU reduced high-risk behavior and promoted their own psychological well-being:

There is a way you do not lose hope when you are HIV infected on CFLU....Personally, I have liked it because it encourages, counsels and one does not lose hope.

IDI, female, 22 years, intervention arm, Kasangati

Before I had multiple sexual partners but now ever since I was taught about risk reduction through health tips, I realized that I was wasting my life then I stopped....

IDI, female, 23 years, intervention arm, Mulago

#### Facility or Service Delivery Factors

##### Strengthened the Clinician-Patient Relationship

The young adults, mainly from IDI, expressed that the tool improved the relationship between health care workers and patients, making the patients feel cared for. The youth said that there was a good relationship and interaction between patients and CFLU doctors. Paying attention to detail, politeness, care, and treatment, as well as offering transport refund by doctors enhanced proper treatment adherence that would lead to health improvement for youth:

There is that element of a good relationship. You see the moment you get used to someone, you try to open up about your challenges; there is that bridging of the gap, that closeness that is created between the two parties.

IDI, male, 24 years, intervention arm, Mulago

##### The Kind Tone of Voice of the System

Most participants from both sites perceived that the tone of voice and the wording used through the system was kind and conveyed friendliness, trust, and care by the health providers. They looked forward to the call that reminded them to take their drugs properly on time:

It’s so soothing and comforting and it makes you feel good. The man says, hallo my beloved, by this time you should have taken your medicine, for sure, you really feel loved and cared for. I really like that voice system.

FGD, female, 18-24 years, intervention, Kasangati

The one speaking speaks in a calm and thankful manner, says “beloved thank you for listening,” and the voice is good.

IDI, female, 23 years, intervention arm, Mulago

Most youth liked CFLU because it taught them how to swallow medicine on time and how to live positively through the routine health tips.

#### Community Factors

##### Confidentiality and Privacy

Most participants from both sites, but mainly from Kasangati, liked confidentiality and privacy through the use of the individualized secret pin code. Young adults were taught how to use the system, especially how to enter their secret pin number, and they found it easy to use:

I was taught, when they call, I have to enter my pin number. Then that is when they will speak. If you don’t put in the pin it won’t talk.

IDI, female, 23 years, intervention arm, Mulago

Reducing the volume of the phone as they listened to voice communication maintained their HIV status privacy, as one participant said:

I reduce the volume of my phone, even if someone notices me answering the call, they can’t know what I’m up to, and they may even ask, eh, you have received a call, how come you are not talking?

IDI, female, 19 years, intervention arm, Mulago

##### Ease of Use for the System

The majority of young adults from both sites found the system easy to use and attributed this to the training or orientation they had received. The system also allows convenient settings for young adults, including individualized pill reminders and time to receive health tips:

They asked me to give them a time that is convenient for me and I choose 9:05 o’clock, and when that time comes, the phone rings to remind me to swallow my medicine.

IDI, female, 19 years, intervention arm, Mulago

### Components of CFLU

#### Beneficial Clinic Visit Call Reminders

All young adults described CFLU as a call system that emphasized mainly pill reminders, health information tips, clinic appointment reminders, and symptom self-reporting, and they were all useful as far as adherence support was concerned.

Most young adults, but mainly from IDI, said that CFLU enhanced keeping routine clinic appointments that promoted ART adherence. Clinic appointment reminders were considered beneficial for those who misplaced cards or forgot appointments. Clinic visit remainders enabled young adults to plan for payment for transport:

When in a faraway place, so when you get a reminder 2 days before the appointment, you plan for transport.

FGD, female, 18-24 years, intervention arm, Kasangati

Appointment reminders are going to be helpful because I have ever missed an appointment, by the time I checked the card the time had passed, so it’s helpful if am reminded.

IDI, male, 23 years, intervention arm, Mulago

#### Value of Symptom Reporting

Through symptom reporting, young adults from both sites, but mainly from IDI, were able to seek treatment and medical advice for themselves or their child. Young adults reported symptoms such as headache, diarrhea, fever, chest pain, abdominal pain, cough, and constipation:

I saw its advantage when I fell sick...CFLU is responsible, when you report your symptom such as cough or fever. You get better or advice and you are told to seek medical attention if the symptom worsens....

FGD, female, young adults, 18-24 years, intervention arm, Kasangati

I had a running stomach one time and a fever...Then I pressed the option for one with a symptom. The doctor called me and told me to come to the clinic the next day and they diagnosed me.

IDI, female, 19 years, intervention arm, Mulago

However, delayed responses from health care workers were a problem for most young adults after self-reporting a symptom. By the time the doctor called back, the symptoms often had disappeared. They recommended that the doctor should call back within 20-30 minutes:

I had a headache, I reported in the night and the doctor called back in the morning but when she called I had swallowed some pain killers.

FGD, female, 18-25 years, intervention arm, Kasangati

#### Use of Health Tips Call

Access to information through health tips increased patient knowledge in young adults from both sites, but mainly IDI. The most frequent health tips selected were positive living, HIV, nutrition, avoidance of risky sexual behaviors such as having multiple partners, alcohol and drug abuse, breastfeeding options, and prevention of mother-to-child transmission. Few young adults reported the fear of stigma and discrimination that was addressed in the health tips messaging:

Initially, at home, it was worse; I suffered low self-esteem due to HIV, as a result, I used to keep everything to myself because I did not want my family members to see me take HIV medication and point fingers at me. I feel I am now a free person after listening to the health tips on avoiding stigma. Call for life reminds me to take my drugs without any body noticing.

IDI, male, 19 years, intervention arm, Mulago

The belief that antiretroviral therapy (ART) cause undesirable side effects that may affect drug adherence was reported by most youth at IDI. Through health tips, young people received basic ART education:

When you have just started drugs let’s say within a month ...one may get dizziness and begin noticing changes..., when they educate us before taking drugs they tell us, expect side effects. You may expect weird dreams, dizziness, that’s why they say you have to choose your appropriate time.

IDI, female, 22 years, intervention arm, Mulago

Majority of young adults attributed good health outcomes to these health tips. For example, one young woman who recently delivered an HIV-negative baby said:

If you take drugs properly without missing, you reduce the chances of infecting the baby. I personally gave birth to a negative baby; I took drugs from here.

IDI, female, 22 years, intervention arm, Kasangati

However, few young adults felt there could be more variation in the health tips offered or that the tips were not relevant to them:

They should not give the same health tips every day, they should ask one, if you want to listen to tips about relationships, you press 1, if you want about pregnancy, then you press that option.

IDI, female, 22 years, intervention arm, Mulago

Majority of young adults requested more diverse and additional health information. Positive female partners in discordant relationships requested for more health information about healthy relationships, disclosure in sero-discordancy, and conception in sero-discordant relationships. Textbox 1 summarizes the different requests.

**Health tips requested by young adults to be added to the Call for Life Uganda system.**

**Health tips and illustrative quotes**

- HIV discordancy and relationship advice
  “Let call for life explain to us more about Prophylactic use of ARVS in a discordant relationship. If one is in a discordant relationship and has been on ARVs for a while, when viral load is fine, the doctors still insist on condom use and you are like for sure! For how long?” (Focus group discussion [FGD], female, 18-24 years, intervention, Kasangati)
- Female condom use
  “I want CFL to add tips on how to use female condoms...I totally failed to use them.” (FGD, female, 18-24 years, intervention arm, Kasangati)
- Relationship handling
  “Actually, talk about the relationships. I think C4L can come in and educate when to have a partner and how to identify and choose a rightful partner.” (in-depth interview [IDI], male, 24 years, intervention arm, Mulago)
- Overcoming the stigma and discrimination (how to disclose the HIV status)
  “Teach us how to manage stigma. I have seen some people...who insult the positive ones. They keep saying so and so is sick and many more words...teach us how to respond to other people in case they stigmatize us.” (IDI, female, 19 years, intervention arm, Mulago)
- Sexual maturation (how to manage body odor)
  “Young people these days have bad odours, so call for life can come in and sensitize on how to ensure good body hygiene. Teach us how to manage and prevent that foul smell.” (IDI, male, 24 years, intervention, Mulago)
- Explanation on viral load
  “Personally, I take a while to know about my viral load. They can tell us whether it is high or low and its implications.” (IDI, male, 24 years, intervention arm, Mulago)
- Dangers of alcohol use and drug abuse
  “Advise youth not to take alcohol and drugs. There are some who use cigarettes and marijuana, there is need to teach them avoidance of harmful habits like alcohol especially when one is on drugs” (IDI, female, 24 years, intervention arm, Mulago)
- Drug side effects
  “Teach us when and how to swallow the drugs. If there is any side effect, explain how to manage them. I think that education can be helpful.” (IDI, male, 24 years, intervention arm, Mulago)
- Basic antiretroviral therapy (ART) education and emphasis on ART adherence
  “Re-emphasize ART adherence...if you don’t swallow your pills you will get such and such problems or if you don’t swallow your drugs on time, you will get such and such problem.” (IDI, female, 23 years, intervention arm, Mulago)
- How to overcome low self-esteem, self-pity, and stress management
  “They should teach me how I should conduct myself in public. I have that problem of low self-esteem.” (IDI, female, 19 years, intervention arm, Mulago)
- Income generation activities or value of vocational skills (art and crafts)
  “Teach us value of income generation activities, teach us vocational skills such as Art and crafts, and Candle making to earn a living.” (FGD, female, 18-24 years, intervention, Kasangati)
- Pregnancy and breastfeeding options
  “Pregnant women need antenatal reminders through call for life system. Just the way routine appointment reminders are done by system.” (IDI, female, 22 years, intervention arm, Kasangati)
- Family planning
  “They should teach us about family planning...how it works some of us who want to access family planning. We were told they have to first check our blood to determine the appropriate method...personally I was on FP but I got an unintended pregnancy.” (IDI, female, 22 years, intervention arm, Mulago)
- Herbal treatment (risks and benefits)
  “Add tips like, you don’t have to use herbal remedies educates me about the risks and benefits of herbs that I was ignorant about.” (IDI, female, 23 years, intervention arm, Mulago)
- Good nutrition
  “You can decide that today I want good nutrition tips and they tell you the food categories that will improve the CD4.” (IDI, male, 19 years, intervention, Kasangati)

#### Daily Pill Reminder Call

Youth, mainly from Kasangati, found that the twice daily remainder call that came through at an agreed specific time was found to be useful in promoting adherence:

What I love the most is the pill reminders because before I joined, I used to take when it’s already past the time, but now I take my drugs on time. That’s what I like most about it.

IDI, female, 22 years, intervention arm, Kasangati

### Barriers to and Challenges of the CFLU System

#### Technical Issues With the System

IDIs and FGD elicited challenges related to the tool. According to most young adults, these were mainly the temporary technical problems that they faced during an upgrade to a newer version. This led to interruptions in calls and the system calling beyond the time scheduled.

Youth from both sites, mainly those from Infectious Diseases Institute Clinic, reported blocked pin codes, the failure of the system to complete outbound health tips, and poor pin recognition (nonresponse after entering a pin code). Most youth reported that the irregularities or inconsistencies of the system were frustrating:

...it was recently messed up a bit. I had stopped picking up the phone calls because every time the call comes through and I put in the pin number, it goes off.

FGD, female,18-24 years, intervention arm, Mulago

#### Poor Access to Mobile Phones

Lost phone problems and battery issues made it difficult for some young people to access CFLU:

There was a time when my phone was stolen which required me to buy another replacement. I was off air for one month. I found it a problem because the system was helpful...

IDI, male, 19 years, intervention arm, Mulago

#### Fear of the HIV-Related Stigma

There was a shared perspective from IDIs and FGD young adults from both sites about the fear of stigma and discrimination associated with HIV. The perception that HIV is a fatal disease associated with promiscuity led to low self-esteem and poor adherence among youth:

Us youths...are shy...proud and boastful with a lot of pride. We don’t want others to know we are positive. If they receive call and accidentally insert the pin code...if accidentally the phone is in loud it can be shameful, one can stop medication...that leads to opportunistic infections.

IDI, male, 19 years, intervention, Kasangati

Youth fear to be stigmatized. For instance, I had a friend but once she found out about my status, she stopped sharing food with me on the same plate, drinking from the same cup, because she did not want to catch HIV.

IDI, 24 years, female, intervention, Mulago

Majority of young adults from the interviews feared being seen taking medicine or answering or responding to a CFLU call because it would be associated with HIV. Few youth feared to be found by their peers or neighbors during their routine clinic visits. Moreover, a few young adults complained of noisy pill tins because they attracted attention. They also worried about their friends spreading rumors about their HIV status:

For me I had a bad chance, the people I found were very ill, I asked for Wednesday, that’s the day, I found that lady who spread rumours. I felt small I was not comfortable even at my work place it still affects me.

FGD, female, 18-24 years, intervention, Kasangati

People pin point at us; the youth, so we fear to take pills. The people may be in the surroundings be it at work or at home, someone may come and rub it your face that you are HIV+. It has happened to me before and I stopped taking my pills.

IDI, 24 years, female, intervention arm, Mulago

### Suggestions and Recommendations From Youth to Improve CFLU

#### Promote Peer Support Meeting

Although there was a positive response to the tool, most young people provided suggestions to improve CFLU. They requested extra support to scale up the CFLU system, such as promotion of peer support meetings among youth with HIV infection on CFLU. This would help them break stigma trends by sharing experiences and attaining peer mentorship. Majority of youth said that peer support meetings would promote youth’s economic capacity to become financially independent. This could be led by empowering them to start an income generation activity as a way of addressing some of the adherence challenges:

Teach us vocational skills like arts and crafts, through peer support meetings we could be given vocational skills of which we can learn to make products for sale to earn a living...CFLU could help and train us who can learn different skills...for transport to hospital and self-care.

IDI, 22 years, intervention arm, Kasangati

#### Community Sensitization About HIV and CFLU

Young adults, mainly from Kasangati, suggested community sensitization about HIV to create awareness. They said extensive community sensitization about HIV reduced the stigma associated with HIV, which could subsequently make it easier for youth to adhere perfectly to their drugs:

...get a day and invite all people whether positive or not to retest for HIV then enrol them on call for life.

IDI, female, 22 years, intervention arm, Kasangati

#### Establish Youth-Friendly HIV Services and Scale Up CFLU

Youth requested for additional health tips, setting up of a designated space and staff for CFLU youth, resolving technical issues, and supporting youth to overcome stigma and discrimination, as summarized in Textbox 2 and Table 3.

**Suggestions by young adults to improve the use of the Call for Life Uganda (CFLU) system.**

**Suggestions and illustrative quotes**

- Encourage young people to maintain the same phone line
  “Many young adults change their phone lines and fail to inform the health providers about it. Tell them stick to one line or in case they change, they should inform the health providers about the new phone lines so that they don’t miss the call.” (Focus group discussion [FGD], female, 18-24 years, intervention arm, Kasangati)
- Resolve technical errors
  “In order to maintain the standard, ensure the call for life staff resolve the technical issues about the system. Staff should ensure the daily calls come through and call at the agreed time. For instance, I take my pills at 10pm but they call me 30 minutes later daily.” (In-depth interview [IDI], female, 24 years, intervention arm, Mulago)
- Make CFLU appealing to young people and explain the benefits
  “Us young people need a TV and games at the clinic waiting areas to occupy us when we arrive as we wait for the call for life doctors to welcome us, health educate us and treat us.” (FGD, female, young adults, 18-24 years, intervention arm, Kasangati)
- Scale up CFLU to all adults and youth in other facilities
  “First, enrol all the adults and young people on call for life system so that they can benefit from it...when they use the system it will help remind them to take their daily medicine on time.” (IDI, male, 19 years, intervention arm, Kasangati)
- Create champions of CFLU to influence other youth to adopt CFLU
  “Organise a day for young people on call for life...to share experiences with fellow youth who are not experienced on call for life system, us on the system are the champions. We can learn from each other about adherence, avoidance of self-pity and benefits of ART...Another thing it is encouraging for us young people when we associate with fellow peers, we can influence youths to join the system. (IDI, female, 19 years, intervention arm, Kasangati)
- Establish a designated space and day for CFLU
  “We need a designated space where we can wait. It is a big challenge for us when we come here. We do not have a call for life premise and usually we met the wrong doctors when we come here. You hear young people say, there is a doctor who called me; I don’t know how to locate the doctor.” (FGD, female, 18-24 years, intervention arm, Kasangati)
- Establish a designated staff to handle young adults enrolled on CFLU at Kasangati Health facility.
  “The call for life staff people should let us know the focal person to talk to when we come here...It’s by luck to find the right doctor and room to go to...we should get a permanent call for life space, so that we know that once we come we go right away to that place or room, Instead of wasting time looking around.” (FGD, female, 18-24 years, intervention arm, Kasangati)
- Sensitize all patients about the CFLU system and do it as a team—doctors, nurses, and patients. Make announcements on television and radio
  “You have to work as a team, doctors, nurses, counselors, tell the patients about the benefits of the system. If you sensitise more people on TV or radio, very many people will come up and join the system. It’s just about creating awareness regarding how the system works and its related benefits.” (IDI, 24 years, intervention arm, Mulago)
- Support young people to overcome the stigma
  “Teach us (young people) how to overcome stigma and respond to people who point fingers at us.” (FGD, female, 18-24 years, intervention arm, Kasangati)
- Combine research study appointments with routine general HIV clinic so that patients do not come twice
  “Suggest that doctors Combine study appointments with general clinic so that patients do not come twice. The return appointment for call for life system should be recorded in the system and in the general file...When one comes for call for life clinic visit...prior to return appointment, they should supply drugs once for all to avoid confusion.” (FGD, female, 18-24 years, intervention arm, Kasangati)

**Table 3:** Suggestions and adherence mitigation strategies from young adults through the Call for Life Uganda (CFLU) system.

Subject and factors hindering ART^a^ adherence				Strategies proposed through CFLU
**Personal barrier**				
	**Forgetfulness secondary to:**			Mitigation
		Busy work schedule	Daily pill reminder function	
		School schedules	Daily pill reminder function	
		Sports activities	Daily pill reminder function	
		Dancing and alcohol	Daily pill reminder function	
**Psychosocial**				
	**Nondisclosure of serostatus**			Health tips call; psychosocial support
		Stigma (fear to be seen taking pills and raising suspicion from people)	Health tips on Psychosocial support	
		Mental issues (low self-esteem as a result of having HIV)	Health tips on Psychosocial support	
		Pride or irresponsibility	Health tips on Psychosocial support	
	Misconception about HIV results (go off drugs when they improve and the viral load is undetected)			Counseling
	Lack of psychosocial support			Treatment buddies and psychosocial support
**Pill related**				
	Drug-related side effects, for example, dizziness and big size of the pill			Symptom reporting and management through the CFLU system
	Sound or noise by pill hitting the pill bottles			Repacking of pills
**Socioeconomic status**				
	Missed clinic appointments			Clinic visit call reminders
	Missed clinic appointments because of lack of transport			Joining community drug distribution points

^a^ART: antiretroviral therapy.

## Discussion

### Principal Findings

In this study, we sought to describe the acceptability of a mobile phone support tool to promote adherence to ART in young adults in an RCT. Understanding the acceptability of a mobile phone support tool to promote adherence to ART in young adults was important to enhance adherence intervention strategies. Four themes emerged from interviews with young adults, including the positive attitude toward CFLU, which included improvement of medication adherence, management of problems of forgetfulness, management of stigma-related issues, and psychosocial support. Negative attitudes toward CFLU were reported under barriers to and challenges of the CFLU theme, including technical issues, poor access to mobile phones, fear of stigma, and financial constraints. One theme was about the components of CFLU and suggestions and recommendations from youth to improve CFLU. This study reveals that the CFLU system supports adherence, which is a critical challenge for youth on ART. The most common barriers to ART adherence mentioned by the young adults in this study included forgetfulness, nondisclosure of serostatus, ART-related side effects, stigma, and pill burden. Forgetfulness is a major factor previously pointed out in other mHealth studies as a barrier to ART adherence [23]. Majority of young adults in this study said that CFLU resolved most of the adherence challenges such as forgetfulness, missed pills and clinic visits, and fear of stigma.

Fear of stigma and discrimination associated with HIV was reported as a challenge to CFLU, which is similar to previous mHealth studies [24]. Health tips emphasized the importance of disclosure to avoid stigma that would affect drug adherence. Patients suggested the desire for CFLU to continue educating youth about HIV and how to overcome stigma through continuous health tips.

Results revealed that CFLU resolved most of the barriers to adherence, such as forgetfulness. A major barrier to ART adherence was also tied closely with daily routine, as reported in some other study [25]. There is a need to make CFLU appealing to the youth so that it can be scaled up to eradicate barriers to ART initiation and adherence, such as fear of disclosing HIV status to partners, drug-related factors (side effects and the big size of the tablet), and HIV stigma [26].

This is in agreement with mHealth interventions that were first deployed in noncommunicable diseases and later used in infectious diseases [27]. Evidence reviews suggest that mHealth interventions delivered in low-income and middle-income countries can be effective in improving health outcomes for people living with chronic diseases [28]. A systematic review of mHealth interventions for monitoring chronic disease by Watkins et al [29] found articles on the monitoring of hypertension, stroke, and people living with HIV from Kenya, Pakistan, Honduras, Mexico, and South Africa. The 6 components of mHealth found in all 4 interventions included reminders, patient observation of health state, motivational education or advice, provision of support communication, targeted actions, and praise and encouragement [29]. Communicating with young adults about health issues and adherence and explaining the possible side effects are important in enhancing their informed decision making.

Concerns about CFLU reported ranged from technical issues, poor access to mobile phones, fear of stigma, and financial constraints. The disadvantages of CFLU reported by young adults were similar to those reported in other studies that reported the cost and convenience of SMS text message, given that its low cost is well suited for supporting the treatment of conditions managed over extended periods compared with interactive voice calls [23]. In the WelTel Kenya 1 trial, SMS intervention was considered inexpensive, and each SMS costed approximately US $0.05, equivalent to US $20 per 100 patients per month, and follow-up voice calls averaged US $3.75 per nurse per month [19]; however, SMS can only be applicable in a population with some literacy [30]. One study in Zanzibar showed that behavioral change was significantly higher with pushed SMS enrollees than with voice messaging enrollees [31]; this was not assessed in our study. However, of the 300 young adults enrolled in the CFLU mHealth intervention arm, only 2 opted for SMS text messaging and were not among the young adults.

Lost phone problems and battery issues made it difficult for some young people to access CFLU, and few young adults preferred messages that they could retrieve after restoring the phone battery. Similar studies report that message delivery rates are far more successful among SMSs than among voice enrollees. Pushing voice messages to clients with personal phones is a complex process that requires the client to answer the phone at the time of delivery, whereas SMS messages can be delivered at any time, including when the phone is turned off. This is the major reason that SMS messages pushed to personal phones have a higher delivery success rate than voice messages [32]. The disadvantage of SMS text messaging reported by other studies includes breaches of confidentiality, and the majority of the patients have a fear of revealing their HIV serostatus, which makes voice calls superior to SMSs unless messages are coded.

The use of the individualized secret pin code enhanced the confidentiality and privacy of most young adults on CFLU. However, blocked pin codes and poor pin recognition of the system hindered the use of the system for most young adults. In a similar study in western Uganda, pin-protected messages reduced the odds of clinic return, as the use of pins was a challenge; hence, they often missed the message alerting them for clinic appointments [30]. Our adolescents advised empowering youth to overcome stigma and setting up a customer help desk at the health facility to resolve technical issues related to CFLU. Other studies suggest the involvement of end users during the development of mHealth apps.

### Limitations

At the time of these interviews (September 2017), the CFLU system was experiencing technical issues following a newer software release; therefore, the calls kept dropping for both outbound and inbound calls. This was resolved first by contacting the software developer and consultant, halting calls, and temporarily turning off the system until the causes for technical issues were investigated and resolved. Automated alerts were developed to notify the principal investigator, the study coordinator, and the information system team on system errors.

During the same period, there was a national deadline by the Uganda Communication Commission to register all phone SIM cards, and some phones were cut off during this period. Young adults bought newly registered phone lines for CFLU.

### Conclusions

The CFLU system can support adherence, despite some of the temporary technical issues. Enhancing adherence to ART using the CFLU system addresses the challenges reported by young people. The CFLU system is user friendly, acceptable, and a feasible strategy to monitor and improve adherence of patients in resource-limited settings.

## Appendix

Multimedia Appendix 1 The call flow from the system to the end user, with functionalities for daily pill reminder calls, clinic visit reminders, health tips, and the interactive guide and options until a call ends.

Multimedia Appendix 2 Flowchart of young adults involved in the qualitative study.

## Abbreviations

ART: antiretroviral therapy
CfL: connect for life
CFLU: Call for Life Uganda
FGD: focus group discussion
IDI: in-depth interview
IVR: interactive voice response
mHealth: mobile health
RCT: randomized controlled trial
