# Supplementary figures and images for: Acceptability of a Mobile Phone Support Tool (Call for Life Uganda) for Promoting Adherence to Antiretroviral Therapy Among Young Adults in a Randomized Controlled Trial: Exploratory Qualitative Study
Source: JMIR Mhealth Uhealth. 2021 Jun 14;9(6):e17418. doi: 10.2196/17418 (PMC8240800; doi:10.2196/17418)

***
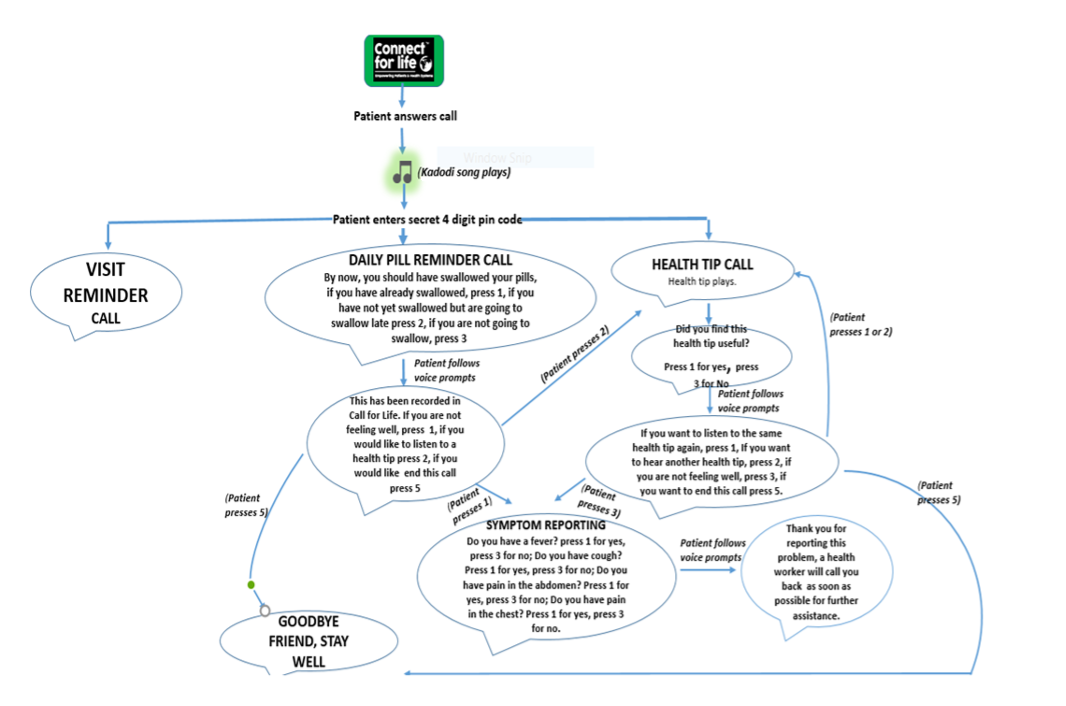
***

Supplement: Multimedia Appendix 1 [file mhealth_v9i6e17418_app1.docx]
