# Supplementary material for: Acceptability of a Mobile Phone Support Tool (Call for Life Uganda) for Promoting Adherence to Antiretroviral Therapy Among Young Adults in a Randomized Controlled Trial: Exploratory Qualitative Study
Source: JMIR Mhealth Uhealth. 2021 Jun 14;9(6):e17418. doi: 10.2196/17418 (PMC8240800; doi:10.2196/17418)
Supplement: Multimedia Appendix 2 [file mhealth_v9i6e17418_app2.docx]

161 Participants from main study assessed for eligibility

82 intervention: 78 Standard of care

82 participants from intervention purposively selected for qualitative

65 Intervention participants excluded

- 37 slightly above age for young adults
- 28 Declined to participate- committed busy schedule

78 Standard of care participants excluded

17participants eligible

- 11 in-depth interviews
- 1 FGD (06 participants)
